# Influence of left ventricular unloading on pediatric post-cardiotomy veno-arterial extracorporeal life support outcomes

**DOI:** 10.3389/fcvm.2022.970334

**Published:** 2022-08-10

**Authors:** Paolo Meani, Roberto Lorusso, Mariusz Kowalewski, Giuseppe Isgrò, Anna Cazzaniga, Angela Satriano, Alice Ascari, Mattia Bernardinetti, Mauro Cotza, Giuseppe Marchese, Erika Ciotti, Hassan Kandil, Umberto Di Dedda, Tommaso Aloisio, Alessandro Varrica, Alessandro Giamberti, Marco Ranucci

**Affiliations:** ^1^Cardio-Thoracic Surgery Department, ECLS Centrum, Heart and Vascular Centre, Maastricht University Medical Centre (MUMC), Maastricht, Netherlands; ^2^Cardiovascular Research Institute Maastricht (CARIM), Maastricht University, Maastricht, Netherlands; ^3^Thoracic Research Centre, Collegium Medicum Nicolaus Copernicus University, Innovative Medical Forum, Bydgoszcz, Poland; ^4^Department of Cardiothoracic and Vascular Anesthesia and Intensive Care Unit (ICU), Istituto di Ricovero e Cura a Carattere Scientifico (IRCCS) Policlinico San Donato, Milan, Italy; ^5^Department of Anesthesiology and Intensive Care, Ospedale Civile Legnano, Legnano, Italy; ^6^Department of Congenital Cardiac Surgery, Istituto di Ricovero e Cura a Carattere Scientifico (IRCCS) Policlinico San Donato, Milan, Italy

**Keywords:** ECLS (VA), congenital hear disease, LV unloading, pediatric, post-cardiotomy cardiogenic shock

## Abstract

**Background:**

The effectiveness of veno-arterial extracorporeal life support (V-A ECLS) in treating neonatal and pediatric patients with complex congenital heart disease (CHD) and requiring cardio-circulatory assistance is well-known. Nevertheless, the influence of left ventricle (LV) distension and its countermeasure, namely LV unloading, on survival and clinical outcomes in neonates and children treated with V-A ECLS needs still to be addressed. Therefore, the aim of this study was to determine the effects of LV unloading on in-hospital survival and complications in neonates and children treated with V-A ECLS.

**Methods:**

The clinical outcomes of 90 pediatric patients with CHD under 16 years of age supported with V-A ECLS for post-cardiotomy cardiogenic shock (CS) were retrospectively reviewed in relationship with the presence or absence of an active LV unloading strategy.

**Results:**

The patient cohort included 90 patients (age 19.6 ± 31.54 months, 64.4% males), 42 of whom were vented with different techniques (38 with atrial septostomy (AS) or left atria cannula, two with cannula from LV apex, 1 with intra-aortic balloon pump (IABP), and one with pigtail across the aortic valve). The LV unloading strategy significantly increased the in-hospital survival (odds ratio [OR] = 2.74, 95% CI 1.06–7.08; *p* = 0.037). On the contrary, extracorporeal cardiopulmonary resuscitation decreased the related survival (OR = 0.32, 95% CI 1.09–0.96; *p* = 0.041). The most common complications were infections (28.8%), neurological injury (26%), and bleeding (25.6%). However, these did not differently occur in venting and no-venting groups.

**Conclusion:**

In pediatric patients with CHD supported with V-A ECLS for post-cardiotomy CS, the LV unloading strategy was associated with increased survival.

## Introduction

The effectiveness of veno-arterial extracorporeal life support (V-A ECLS) in supporting neonatal and pediatric patients with post-cardiotomy shock, following correction of complex congenital heart disease (CHD), is well-established (1). Although V-A ECLS is able to unload the right ventricle, the effect of retrograde flow in the aorta toward the left ventricle (LV) is one of the most important concerns in this setting. Indeed, mainly in the presence of severe myocardial dysfunction, the V-A ECLS-related LV afterload increase may not be overcome by the LV (2). This may lead to LV dilatation, increased left atrial pressure, and pulmonary edema. In addition, LV overload increases wall stress and myocardial oxygen consumption, jeopardizing ventricular recovery. In case of severe overload, the aortic valve may remain constantly closed causing blood stasis and thrombi formation in the LV (3).

Left heart overload in pediatric patients managed on V-A ECLS may be mainly addressed by atrial septostomy (AS) (4), although alternative techniques may also be applied (5).

Most of the reported experience of LV decompression in pediatric patients supported with V-A ECLS consists of case series and limited populations (6, 7). The few largest published studies were able to demonstrate that adequate LV decompression can only prevent the abovementioned-related complications (8, 9). Given the limitations of these studies, however, LV unloading strategy in pediatric patients has never been specifically addressed and associated with improved ECLS in-hospital survival (9, 10).

We aimed to determine the effects of LV unloading on in-hospital survival and complications in neonates and children treated with V-A ECLS in a referral center for pediatric cardiac surgery.

## Methods

Between December 2010 and January 2020, a total of 115 patients received V-A ECLS support in our pediatric intensive care unit (ICU). Patients with complex anatomy were excluded. A complex anatomy was defined as the presence of mixing physiology at the atrial or ventricular level with a documented shunt between right and left circulation or when this shunt could not be excluded. Therefore, this could not allow to judge the effect of LV venting.

The analyzed patients were <16 years of age, and the mean age was 17.3 ± 31.54 months. Notably, 64.4% of patients were males. The indication for V-A ECLS was the evidence of low cardiac output syndrome (defined as clinical evidence of relative hypotension or tachycardia without severe signs of hypoperfusion; systolic blood pressure <90 mmHg or mean arterial pressure <60 mmHg or >30 mmHg drop from baseline and/or pulse ≥100 beats per minute and/or, if hemodynamics done, cardiac index ≥2.2 L/min/m^2^ with lactates between 2 and 3 mmol/L), cardiogenic shock (CS; defined as clinical evidence of relative hypotension with signs of severe hypoperfusion that requires interventions beyond volume resuscitation to restore perfusion; systolic blood pressure <90 mmHg or mean arterial pressure <60 mmHg or >30 mmHg drop from baseline and drugs/device used to maintain blood pressure above these targets, if hemodynamics done, cardiac index <2.2 L/min/m^2^ with lactates >3 mmol/L), or extracorporeal cardiopulmonary resuscitation (E-CPR) (defined as the implantation of VA-ECLS in a patient who experienced a sudden and unexpected pulseless condition attributable to cessation of cardiac mechanical activity).

We retrospectively reviewed the major clinical outcomes, including survival and complications, in relationship with the occurrence of an active (therapeutic) LV unloading strategy.

### Techniques of left ventricular venting

Decompression procedures were performed in the operative room immediately after V-A ECLS initiation. The LV unloading strategy was set at the discretion of the local Heart Team (including the intensivist, the cardio-surgeon, and the anesthesiologist) each time such a procedure was considered necessary. The main criteria for this decision-making process were the hemodynamic status, the ongoing inotropic support, and the related degree of LV distension/dysfunction, defined with an echocardiographic evaluation (depressed LV ejection fraction, LV dilatation with/without “smoke-like” effect, and increased LV filling pressure). A venting strategy was used in patients whenever there was evidence of poor decompression of the left side of the heart. If left-sided structures were distended, a low-moderate dose of epinephrine infusion (<0.1 μg/kg/min) was started to improve the contractility and LV ejection. LV decompression was achieved in different locations in our center: (1) left atrium: either with a direct insertion of a venting cannula through the superior pulmonary vein or through AS, (2) LV: either with a cannula draining blood from the LV apex or pigtail catheter across the aortic valve, and (3) aorta: intra-aortic balloon pump (IABP). The decision among the available unloading techniques was based on three main criteria, namely, clinical scenario, patient anatomical features, and surgical expertise.

The unloading effect was carefully monitored during the following ICU stay through serial clinical, echocardiographic, and instrumental (X-ray) evaluations.

### Atrial septostomy

An atrial septal fenestration (2.5–3.5 mm according to the patient's weight) was surgically performed in the overwhelming majority of the patients, leading to left-right communication. Differently and less frequently, according to the clinical scenario and the applicable cannula size, we also placed a curved venous cannula (Edwards Lifescience TF010-090, Pacifico cannula 8–10 Fr) in the left atrium as a drainage cannula through the superior pulmonary vein.

### Left ventricular apex venting and pigtail across the aortic valve

These techniques can draw directly from the LV. The LV venting cannula needed to be carefully implemented from the LV apex in the operation room. In contrast, a guide wire was first used to cross the aortic valve, allowing the 6 F pigtail catheter to be advanced over it into the LV in a small size neonate, weighing <3 kg. Patients were carefully observed with electrocardiographic and hemodynamic monitoring.

### Intra-aortic balloon pump implantation

The IABP implementation in a pediatric patient should be carefully evaluated and performed. The choice of the insertion site and balloon length, according to the related guidelines (11), was crucial to avoid complications, such as cerebrovascular accidents and renal and mesenteric ischemia. We used to implant IABP only in pediatric patients weighing above 40 kg. Therefore, the contralateral femoral artery of the V-A ECLS cannulation site was used for balloon placement (Seldinger method). If feasible (no major resistance at IABP passage through the small skin incision), a sheathless technique was used to reduce the incidence of leg ischemia. The tip of the balloon was placed 1 cm distal to the junction with the left subclavian artery, as assessed by echocardiographic assessment and by a mobile chest X-ray system at the bedside. Either the electrocardiogram or the aortic blood pressure curve was used as a trigger; for the electrocardiogram, the descending section of the R wave (representing the closing of the aortic valve) was used to calibrate the counter-pulsation interval, with an IABP ratio of 1:1.

### Primary and secondary endpoints

In-hospital patient survival was the primary endpoint investigated to assess the effect of LV unloading. Furthermore, the secondary endpoints included the mortality on ECLS and after weaning, as well as all the occurred complications, including liver and kidney end-organ damage (defined as creatinine and bilirubin peaks). Data on adverse events included cerebral injury (stroke, transitory ischemic attack, intracranial hemorrhage, and seizures by electroencephalogram), acute kidney injury requiring continuous renal replacement therapy, hemolysis (defined as increased free hemoglobin level above 50 mg/dl) (12), peripheral vascular damage, infections (defined as positive bacterial, fungal or viral culture, or polymerase chain reaction test), coagulation disorders (either thrombosis or hemorrhage), and ECLS failure (pump or oxygenator failure, or both).

### Statistical analysis

Unless otherwise specified, data are presented as mean (minimum–maximum) or frequency (%). Paired vented/no-vented VA-ECLS data were compared using two-tailed significance *t*-test for independent continuous samples or two-tailed significance chi-square test for categorical variables. The association between the main outcomes and dependent predictors was tested through a binary logistic regression model. Furthermore, based on the binary logistic regression model and depending on the emerged significant variables, the patients were assigned to different groups. Survival assessment included Kaplan–Meier analysis with the log rank test for differences between groups, producing a hazard ratio with a 95% confidence interval. All the statistical tests were performed using computerized packages (SPSS 22.0, IBM, Chicago, IL, and a MedCalc, Ostend, Belgium).

## Results

Among 115 pediatric patients supported with V-A ECLS in our pediatric intensive care unit, 25 patients were excluded according to their complex anatomy. A total of 90 patients were analyzed. All supports were placed in a post-cardiotomy setting. Among them, 26.7% accounted for Dextro-Transposition of the great arteries (d-TGA), 14.4% accounted for pulmonary artery disorders, and 12.2% were represented by other valve diseases. The distribution of CHD is presented in detail in Table 1. In 42 patients, the LV was vented, while the remaining 48 patients were supported with V-A ECLS alone.

**Table 1:** Distribution of congenital heart disease.

**Baseline congenital heart disease**	***N* (Percentage)**
LA aneurysm and Superior Cava Vein in Coronary sinus	1 (1.1 %)
Anomalous pulmonary venous return	4 (4.4%)
Atrioventricular canal (AVC)	5 (5.6%)
AVC	4
AVC and hypoplastic Aortic Arch	1
Combined disorder	6 (6.7%)
Coronary artery abnormalities	6 (6.7%)
Double outlet right ventricle	3 (3.3%)
Aortic arch interruption	1 (1.1 %)
Outflow tract obstruction	1 (1.1 %)
Patent ductus arteriosus	2 (2.2 %)
Pulmonary artery disorders	13 (14.4%)
Pulmonary atresia and Ventricular septal defect	1
Pulmonary atresia, Ventricular septal defect and MAPCAS	8
Pulmonary and aortic stenosis	2
Pulmonary sling	2
Shone syndrome	2 (2.2%)
Dextro-Transposition of the Great Arteries (d-TGA)	24 (26.7%)
d-TGA	12
d-TGA and Atrial or Ventricular septal defect	10
d-TGA and Ebstein	1
d-TGA + pulmonary stenosis	1
Tetralogy of Fallot (TOF)	2 (2.2%)
TOF	1
Tetralogy of Fallot and Pulmonary atresia	1
Truncus arteriosus	2 (2.2%)
Truncus arteriosus	1
Truncus arteriosus and aortic stenosis	1
Valve disease, other than pulmonary valve	11 (12.2%)
Others	7 (7.8%)

MAPCAS, Major Aortopulmonary Collateral Arteries, LA, left atrium.

### Demographic and clinical features

The two groups didn't present any significant differences in terms of age, weight, risk adjustment for congenital heart surgery method 1, and main baseline features such as kidney or liver function. Patients who received a venting strategy had a higher occurrence of either systemic or pulmonary hypertension (systemic hypertension: no-venting 18.3% vs. venting 45.2%, *p* = 0.021; pulmonary hypertension: no-venting 16.3% vs. venting 23.8%, *p* = 0.013). E-CPR occurrence did not differ between the two groups (no-venting 26.7% vs. venting 23.8%, *p* = 0.759). All demographic and clinical features are shown in Table 2.

**Table 2:** Demographic and clinical features.

	**No Venting** **(*N* = 48)**	**Venting** **(*N* = 42)**	***p*-value**
**Demographic**			
Age, days	693.9 ± 1167.7	476.6 ± 835.0	0.316
Male	69.4%	59.5%	0.326
Weight, kg	9.4 ± 9.5	8.0 ± 6.8	0.427
Height, cm	74.3 ± 28.9	72.7 ± 24.5	0.773
BSA	0.4 ± 0.3	0.4 ± 0.2	0.540
**Comorbidities**			
Previous CVA	4.3%	7.1%	0.555
Hypertension	Mild: 16.3% Moderate: 2.0%	Mild: 38.1% Moderate: 7.1%	0.021
Pulmonary Hypertension	Mild: 4.1% Moderate: 12.2%	Mild: 21.4% Moderate: 2.4%	0.013
Cyanotic	53.1%	45.2%	0.457
**Surgery**			
Open chest	83.7%	90,5%	0.339
RACHS I	3.2 ± 0.9	3.2 ± 0.5	0.691
AoR	2.0%	2.4%	0.912
MVR	4.1%	4.8%	0.875
CABG	2.0%	0%	0.875
**Arteriopathy**			
Inferior Arms	2.0%	0%	0.875
Thoracic Aorta	10.2%	4.8%	0.331
**Clinical features**			
EF, %	41.2 ± 15.8	37.1 ± 18.4	0.266
Creatinine, mg/dl	0.5 ± 0.4	0.5 ± 0,4	0.647
Bilirubin, mg/dl	2.8 ± 3.8	1.9 ± 3.1	0.275
Lowest Hb, g/dl	9.3 ± 1.4	9.2 ± 1.2	0.786

AoR, aortic regurgitation; BSA, body surface area; CABG, coronary artery bypass graft; CVA, cerebral vascular accident; EF, ejection fraction; Hb, Hemoglobin; MVR, mitral valve regurgitation; RACHS-1, risk adjustment for congenital heart surgery method 1.

### Veno-arterial extracorporeal life support features

Different venting techniques were used as LV unloading strategies in our patients. The majority received venting from the left atrium (*N* = 38, 90.5%), either with a venting cannula (*N* = 8), or through AS (*N* = 30). Three patients were directly vented in the LV (*N* = 3, 7.15%), two children through a draining cannula from the LV apex (*N* = 2) and one neonate with a pigtail catheter across the aortic valve (*N* = 1). Finally, according to the body size, one IABP (*N* = 1, 2.35%) was placed as a venting strategy.

Technically, V-A ECLS duration was not different between vented and not vented supports. Peripheral cannulation was used more frequently in the no-venting group (no-venting 18.4.% vs. venting 4.8%, *p* = 0.047). Regarding the peripheral setting, the favorite arterial cannulation sites in the peripheral mode were carotid artery and femoral artery, respectively, whereas femoral and jugular veins were the most common sites for the venous cannula. Finally, all central V-A ECLS was placed using the aorta and the right atrium as implantation sites. Bivalirudin was the chosen anticoagulation strategy in 54.2% of no-vented ECLS and 47.6% of vented V-A ECLS (*p* = 0.535). Table 3 describes the V-A ECLS features.

**Table 3:** V-A ECLS and LV Unloading features.

	**No venting** **(*N* = 48)**	**Venting** **(*N* = 42)**	***p*-value**
**V-A ECLS features**			
E-CPR	26.7%	23.8%	0.759
ECLS, hours	134.5 ± 87.4	142.4 ± 86.6	0.696
Peripheral cannulation	18.4%	4.8%	0.047
Arterial Cannula	Aorta: 81.6% Femoral Artery: 6.1% Carotid Artery: 12.2%	Aorta: 95.2% Femoral Artery: 2.4% Carotid Artery: 2.4%	0.132
Cannulation Mode	Direct: 100% Distal reperfusion: 6.1%	Direct: 95.2% Distal reperfusion: 2.4% Vasc prosthesis: 2.4%	0.388
Venous Cannula	Atria-PV: 81.6% Femoral Vein: 8.2% Jugular Vein: 10.2%	Atria-PV: 95.2% Femoral Vein: 2.4% Jugular Vein: 2.4%	0.139
Bivalirudin	54.2%	47.6%	0.535
**Vent location/strategy**			
LA/ AS or LA cannula		38 (90.5%)	NA
		AS (30)	
		LA cannula (8)	
LV/ LV cannula		2 (4.8%)	
Aorta/IABP		1 (2.35%)	
LV/Pigtail across AV		1 (2.35%)	

AS, atrial septostomy; AV, aortic valve; IABP, intra-aortic balloon pump; LA, left atrium; LV, left ventricle; Vasc, vascular; PV, pulmonary vein.

### Primary and secondary outcomes

The primary and secondary outcomes are shown in Table 4. All major complications occurring on V-A ECLS such as stroke, acute kidney injury, and bleeding did not show any significant difference between groups. Regarding in-hospital mortality, patients who were vented on V-A ECLS showed a significantly higher survival at discharge (no-venting 51.0% vs. venting 73.8%, *p* = 0.026), although deaths on V-A ECLS did not differ between the two groups, with a higher post-weaning death rate in no-venting V-A ECLS patients (no-venting 22.4% vs. venting 7.1%, *p* = 0.398).

**Table 4:** Primary and secondary outcomes.

	**No venting (*N* = 48)**	**Venting (*N* = 42)**	***p*-value**
**Primary outcomes**			
Deaths on ECLS	26.5%	19.0%	0.398
Deaths after weaning	22.4%	7.1%	0.044
In-hospital Survival	51.0%	73.8%	0.026
**Secondary outcomes**			
Infections	34.6 %	22.2%	0.443
Cerebral Injury	21.7%	31.0%	0.144
CRRT	20.4%	22.0%	0.902
Hemolysis	17.1%	11.8%	0.855
Thrombosis	14.6%	14.3%	NS
Bleeding	22.9 %	28.6%	0.397
DIC	0%	4.8%	NS
Bleeding, ml	809.5 ± 1383.4	1000.0 ± 1303.0	0.512
Vascular damage	2.1%	7.1%	0.245
ECLS failure	14.4%	11.9%	0.646
**Organ damage**			
Peak creatinine, mg/dl	0.8 ± 0.7	0.9 ± 0.5	0.540
Peak Bilirubin, mg/dl	5.5 ± 5.8	5.4 ± 10.2	0.925

DIC, disseminate intravascular coagulopathy; CRRT, continuous renal replacement therapy; ECLS, extracorporeal life support.

### Predictors of survival

The main predictors of the in-hospital survival were the use of venting strategy and the absence of E-CPR. The venting strategy significantly increased the survival at the discharge by almost three times (odds ratio [OR] = 2.74, 95% CI 1.06–7.08; *p* = 0.037). On the contrary, E-CPR was associated with decreased survival (OR = 0.32, 95% CI 1.09–0.96; *p* = 0.041). These results were adjusted for risk adjustment for congenital heart surgery method 1 class, peripheral cannulation, age, and the presence of pulmonary hypertension, as presented in Table 5.

**Table 5:** Logistic regression model predicting in-hospital survival.

	**Regression coefficient**	***p*-value**	**OR**	**95% CI**	
				**Lowe**	**Upper**
LV Venting	1.008	0.037	2.741	1.061	7.079
RACHS-1 class	−0.016	0.960	0.985	0.534	1.816
Peripheral Cannulation	−0.411	0.615	0.663	0.133	3.290
E-CPR	−1.129	0.041	0.323	0.109	0.957
Age	0.000	0.166	1.000	1.000	1.001
PH	−0.443	0.263	0.642	0.295	1.395

OR, odds ratio; CI, confidence interval; LV, left ventricle; RACHS-1, risk adjustment for congenital heart surgery method 1; E-CPR, extracorporeal cardiopulmonary resuscitation; PH, pulmonary hypertension.

**Figure 1:**
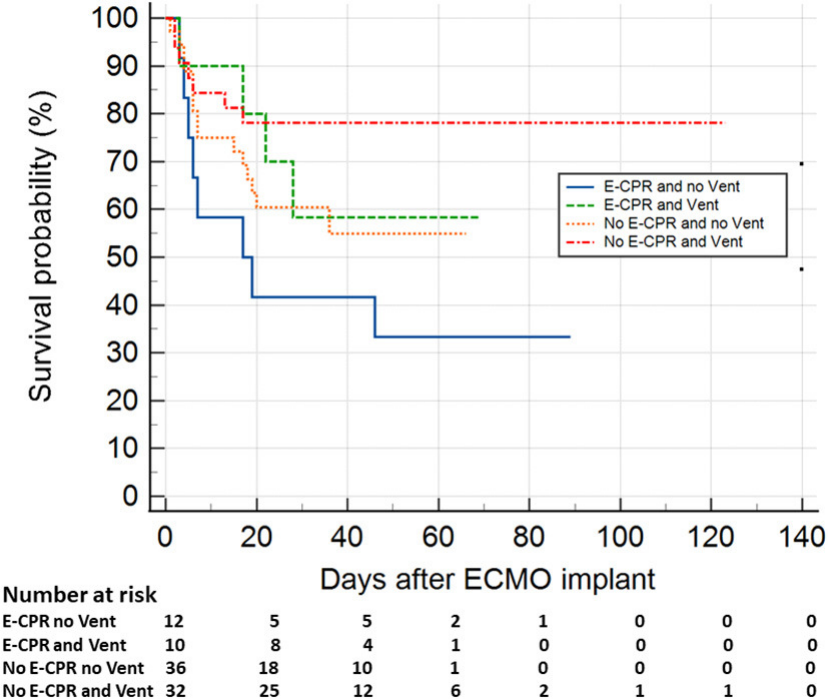
Survival in pediatric patients supported with V-A ECLS, based on the presence/absence of E-CPR and LV unloading strategy. The function showed a significant higher survival (*p* = 0.012) in patients without E-CPR and receiving venting compared to those undergone to E-CPR and who had no LV unloading (hazard ratio 3.6, 95% confidence interval 1.18–11.0). E-CPR, extracorporeal-cardiopulmonary resuscitation; ECMO, extracorporeal membrane oxygenator.

Based on the regression model result, the patients were divided into four groups (E-CPR + no-venting, *N* = 12; E-CPR + venting, *N* = 10; no E-CPR + no-venting, *N* = 36; and no E-CPR + venting, *N* = 32). The survival function (Figure 1) demonstrated a significant difference (*p* = 0.012) between patients with E-CPR and no-venting and patients without E-CPR and receiving venting (hazard ratio of 3.60, 95% CI 1.18–11.0).

## Discussion

To the best of our knowledge, this study represented one of the largest investigations on pediatric populations with CHD supported with V-A ECLS in a post-cardiotomy setting, in association with different LV venting techniques. Furthermore, the complex anatomies that characterized patients referred to our national referral center for pediatric cardio surgery gave us the opportunity to investigate the role of these strategies specifically and adequately in a unique setting.

First of all, in this population of patients with CHD supported with V-A ECLS, we found a rate of major complications compared with other experiences (13).

Our findings demonstrate the high rates of acquired infection and bleeding during pediatric V-A ECLS. Our observed rates are consistent with a recent meta-analysis on post-cardiotomy ECLS in pediatric patients. In fact, Lorusso et al. (13) showed a rate of infections ranging from 3.1 to 50%, while bleeding is highly variable, peaking at 69%.

Neurological injuries were also very common in our children supported with ECLS. In a study by Chow et al. involving 90 patients, only 15 children survived without neurological sequelae (14). On the contrary, in the Extracorporeal Life Support Organization (ELSO) Registry, only 14% had a neurological complication (15). In our population, 13.3% accounted for neonates weighing <3 kg and 24.4% accounted for E-CPR, which are considered well-known risk factors for neurological injury (14). As a consequence, this may lead to a high rate of neurological complications.

Furthermore, one out of the five patients required CRRT. This confirmed that acute kidney injury frequently occurred in pediatric patients on ECLS in a post-cardiotomy setting, ranging from 9 to 78% in the literature (13). All ECLS complications own a negative impact on the survival (16).

Although the rate of complications did not differ in venting and no-venting groups, the in-hospital survival was significantly higher in vented patients. The aim of this study was to determine the effects of LV unloading primarily on in-hospital survival and secondarily on the complications.

Only very few studies have previously evaluated the association of LV unloading with major outcomes in children or neonates supported by V-A ECLS. Choudhury et al. (17) in their retrospective review of the ELSO Registry, revealed an association between left heart decompression and a higher OR of survival in children with myocarditis and dilated cardiomyopathy on extracorporeal membrane oxygenation. On the contrary, Eastaugh et al. (8) did not find a difference in mortality in a single-center experience evaluating the impact of percutaneous left heart decompression in pediatric patients on V-A ECLS. However, this study included a highly heterogenic population, since this consisted of children with CHD mixed with structurally normal hearts. Zampi et al. (18) identified the impact of earlier LV unloading on clinically important outcomes, such as ECLS and mechanical ventilation durations, but it failed to reveal an impact on in-hospital survival.

Despite the lack of consensus in the literature, our study interestingly showed a strong association between LV venting and in-hospital survival rate in pediatric patients supported with V-A ECLS. LV venting is able to guarantee short-term benefits, directly related to the hemodynamic effects, and also middle-long term advantages, mostly linked with myocardial remodeling prevention.

The potential benefits of LV venting have been recently addressed and described in the computational model (19), as well as preclinical (20) and clinical setting (21, 22). When the LV is unloaded, LV mechanical power expenditure is reduced, which minimizes myocardial oxygen consumption and reduces the hemodynamic forces leading to ventricular remodeling (23). As a direct consequence, unloading reduces infarct size and preserves mitochondrial function after ischemia–reperfusion injury (24). Therefore, venting the LV on V-A ECLS may mitigate the acute negative effects of the increased LV afterload generated by retrograde flow Subsequently, the advantages of LV unloading are more related to the prevention of myocardial remodeling, ventricle dilation, and severe residual dysfunction. In fact, stretching of cardiomyocytes induces alterations in multiple intra- and extra-myocyte pathways in parallel, including sarcomere changes, cytoskeletal proteins, and mitochondria (23). In addition, the inflammation initiated by the tissue injury plays an important role, activating matrix metalloproteases primed to receive the increased hemodynamic load force (23). Furthermore, the pressure overload acts synergistically with tissue injury to cause LV remodeling in a mouse model as demonstrated by Weinheimer et al. (25).

In our population, the mortality on V-A ECLS was not significantly different in patients who were vented. In the acute phase, under cardio-circulatory shock conditions of severe end-organ hypoperfusion, the clinical effect of LV unloading might be less appreciable. However, the myocardial protection promoted by the LV unloading seems to be crucial in the subacute phase, namely, after weaning, with a consistently lower mortality in vented V-A ECLS supports during the post-ECLS hospital course.

Among all the available LV venting techniques (5), the choice should be guided by the ongoing clinical scenario, the patient anatomical features, and the operator expertise. First, the pediatric size significantly limits the panel of usable techniques. Second, the clinical scenario in our population strongly favors the surgical LV unloading approach, since all the patients have already been in the operative room (5). Therefore, the overwhelming majority of our pediatric patients were vented in the left atrium, by AS or by placing a venting cannula through a pulmonary vein. Eastaugh et al. also reported left heart unloading in 42 patients supported with V-A ECLS, *via* AS or left atrial venting across the atrial septum (8). All techniques were percutaneous and equally effective. In another monocentric study, Hacking et al. showed left heart decompression in children on central V-A ECLS. They reported 39 cases successfully managed with left atrial venting and only 5 with AS (9).

Our findings confirmed the safety and effectiveness of AS, as previously suggested by the abovementioned experiences (8, 9). This is in contrast with the recent results of the IMPACT registry. Deshpande et al. collected 233 patients who underwent percutaneous AS. This procedure was associated with significant morbidity, including procedural complications (26). On the one hand, this registry recorded data from 55 independent centers whose local expertise might significantly vary. On the other hand, the percutaneous approach might lead to a high rate of procedural complications compared to the surgical septostomy used in our CHD cohort.

**Figure 2:**
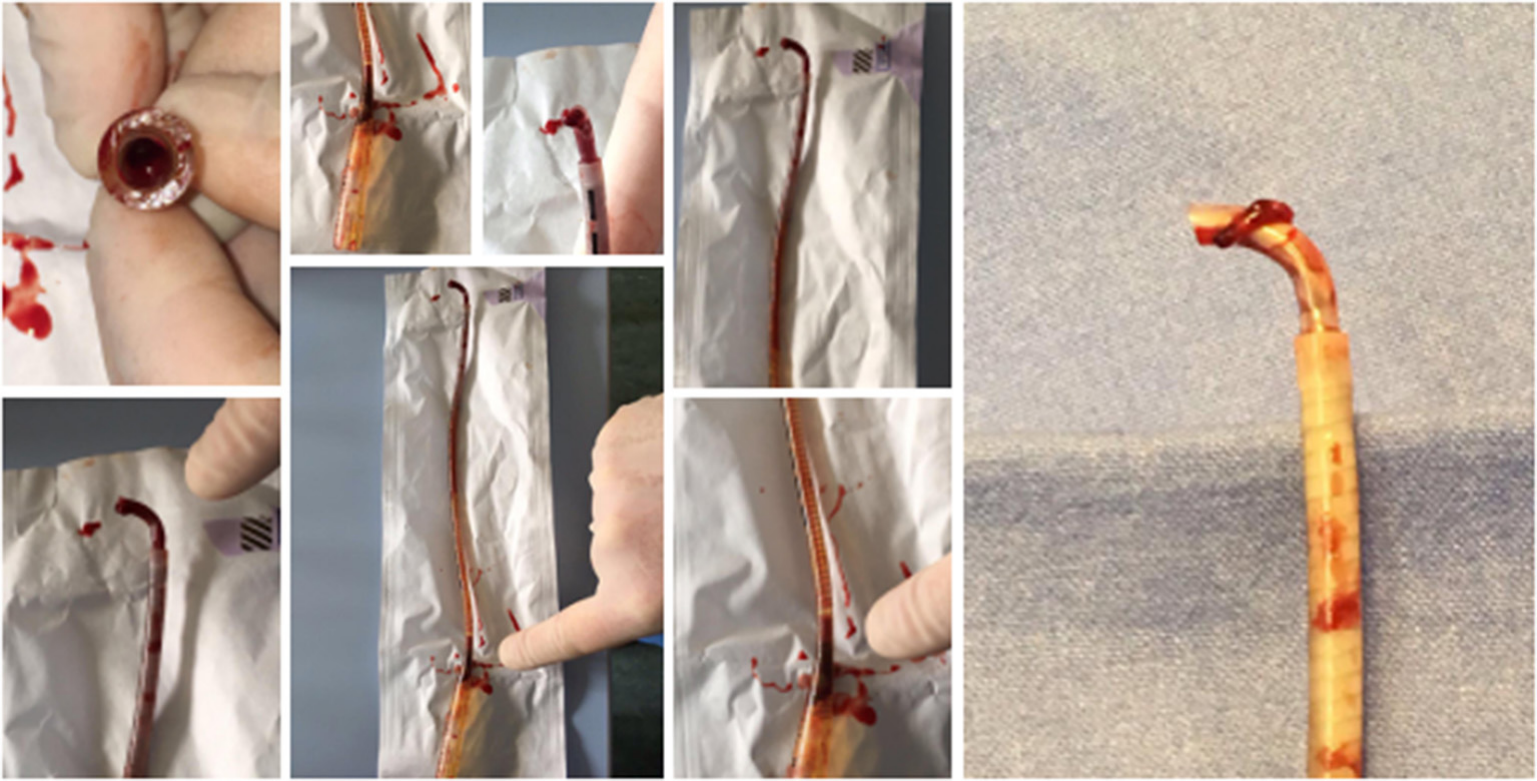
Clots found in the left atrium cannula. The presence of significant thrombi occurred in three cases in this series of patients. This required urgent removal, since the clots obstructed the cannula blood flow. Thereafter, the cannula was promptly removed, and the remaining atrial septum hole left as vent strategy.

Furthermore, the surgical approach may involve small size cannulas inserted either in the left atrium or LV (5). However, we encountered three cases of left atrium cannula thrombosis that required urgent removal, as shown in Figure 2. The cannula was promptly removed and replaced. This concern and other shortcomings regarding left atrium cannula in children, particularly in neonates, were previously reported (8).

To summarize, we consider AS as the first current LV unloading strategy in pediatric post-cardiotomy ECLS, which is also supported by the low frequency of hemodynamically significant residual atrial shunt (8). This strategy avoids the risk of cannula thrombosis, and it is feasible in the surgical setting. Accordingly, Sperotto et al. (4) recently showed that LA decompression, either surgical or percutaneous, independently decreased the risk of in-hospital adverse outcomes in pediatric VA-ECLS who failed to wean from cardiopulmonary bypass.

The impact of LV venting on in-hospital survival was significant in our study. The probability of survival at discharge was almost three times higher in patients undergoing LV venting, despite risk adjustment for congenital heart surgery method 1 class, peripheral cannulation, age, pulmonary hypertension, and E-CPR. The latter was negatively associated with in-hospital survival, as already confirmed in the literature (27).

This result on LV unloading is in accordance with the most recent evidence described in adult-related investigations. Russo et al. (28) in a meta-analysis of 17 observational studies, found an association between LV unloading and decreased mortality in adults with CS treated with V-A ECLS (28). However, the adult population is characterized by several confounding factors (29). As a result, most of the proofs regarding the LV unloading impact in adults have required matched populations (21, 30). Therefore, the impact on the survival found in our pediatric population may underline the real effect of LV unloading in a homogeneous patient group without significant confounding factors, like comorbidities. The pediatric population affected by a primary cardiac disease and common absence of further comorbidities might represent a more appropriate condition to investigate the impact of LV venting on the overall survival. Moreover, the pediatric patients with CHD represent a selected cohort that might have additional benefits from LV unloading compared to other populations.

Additional studies are, however, warranted to further investigate and confirm our findings on the advantage of LV venting on V-A ECLS-related in-hospital survival in pediatric patients.

### Limitations

This study should be considered in the context of some limitations.

First, this is not a randomized controlled trial examining the use of LV unloading during V-A ECLS in the pediatric population. Data are merely derived from an observational retrospective dataset and, therefore, influenced by biases related to this study design.

There were scanty data to investigate survival in relation to the specific CHD or cardiac surgery and LV unloading strategy (i.e., left atrium venting vs. LV apex venting vs. IABP vs. pigtail across the aortic valve) with adequate statistical power. Furthermore, specific etiology of CS was not available for the study analysis. All these factors might temper the found relationship between left ventricular unloading during V-A ECLS and survival.

## Conclusion

In pediatric patients supported with V-A ECLS for CS or cardiac arrest, the implementation of a concomitant LV unloading strategy was associated with predicted higher in-hospital survival. LV unloading should be strongly considered for selected pediatric patients with CHD in a post-cardiotomy setting. Further investigations are urgently needed to better clarify this apparently significant advantage.

## Data availability statement

The raw data supporting the conclusions of this article will be made available by the authors, without undue reservation.

## Ethics statement

Ethical review and approval was not required for the study on human participants in accordance with the local legislation and institutional requirements. Written informed consent from the participants' legal guardian/next of kin was not required to participate in this study in accordance with the national legislation and the institutional requirements.

## Author contributions

All authors listed have made a substantial, direct, and intellectual contribution to the work and approved it for publication.

## Conflict of interest

Author RL was consultant for Medtronic, Getinge, and LivaNova and member of the Advisory Board for Eurosets and Xenios. Author MR received spekaer's honoraria, consultancy fees, and research grants from: Haemonetics Werfen-IL Haemosonics Roche Diagnostics CSL Behring Livanova Medtronic.

The remaining authors declare that the research was conducted in the absence of any commercial or financial relationships that could be construed as a potential conflict of interest.

## Publisher's note

All claims expressed in this article are solely those of the authors and do not necessarily represent those of their affiliated organizations, or those of the publisher, the editors and the reviewers. Any product that may be evaluated in this article, or claim that may be made by its manufacturer, is not guaranteed or endorsed by the publisher.

## Abbreviations

AS: Atrial septostomy
E-CPR: extracorporeal-cardiopulmonary resuscitation
CHD: Congenital Heart Disease
IABP: Intra-aortic Balloon Pump
LV: Left Ventricle
V-A ECLS: Veno-arterial extracorporeal life support.
